# High-flow nasal therapy versus noninvasive ventilation in COPD patients with mild-to-moderate hypercapnic acute respiratory failure: study protocol for a noninferiority randomized clinical trial

**DOI:** 10.1186/s13063-019-3514-1

**Published:** 2019-07-22

**Authors:** Andrea Cortegiani, Federico Longhini, Annalisa Carlucci, Raffaele Scala, Paolo Groff, Andrea Bruni, Eugenio Garofalo, Maria Rita Taliani, Uberto Maccari, Luigi Vetrugno, Enrico Lupia, Giovanni Misseri, Vittoria Comellini, Antonino Giarratano, Stefano Nava, Paolo Navalesi, Cesare Gregoretti

**Affiliations:** 1 Department of Surgical, Oncological and Oral Science (Di.Chir.On.S.), Section of Anesthesia, Analgesia, Intensive Care and Emergency, Policlinico Paolo Giaccone, University of Palermo, Via del vespro 129, 90127 Palermo, Italy; 2 Intensive Care Unit, Department of Medical and Surgical Sciences, University Hospital Mater Domini, Magna Graecia University, Catanzaro, Italy; 3 Pulmonary Rehabilitation Unit, Istituti Clinici Scientifici Maugeri, Pavia, Italy; 4 Pulmonology and Respiratory Intensive Care Unit, S. Donato Hospital, Arezzo, Italy; 5 Emergency Department, “S. Maria della Misericordia” Hospital, Perugia, Italy; 6 Department of Anaesthesia and Intensive Care, University of Udine, Udine, Italy; 7 Emergency Department, “Città della Salute e della Scienza” University Hospital, Torino, Italy; 8 Department of Clinical, Integrated and Experimental Medicine (DIMES), Respiratory and Critical Care Unit, S. Orsola-Malpighi Hospital, Alma Mater University, Bologna, Italy

**Keywords:** High-flow oxygen therapy through nasal cannula, Noninvasive ventilation, Chronic obstructive pulmonary disease, Carbon dioxide, Acute respiratory failure, Intensive care unit, Emergency department

## Abstract

**Background:**

Noninvasive ventilation (NIV) is indicated to treat respiratory acidosis due to exacerbation of chronic obstructive pulmonary disease (COPD). Recent nonrandomized studies also demonstrated some physiological effects of high-flow nasal therapy (HFNT) in COPD patients. We designed a prospective, unblinded, multicenter, randomized controlled trial to assess the noninferiority of HFNT compared to NIV with respect to the reduction of arterial partial pressure of carbon dioxide (PaCO_2_) in patients with hypercapnic acute respiratory failure with mild-to-moderate respiratory acidosis.

**Methods:**

We will enroll adult patients with acute hypercapnic respiratory failure, as defined by arterial pH between 7.25 and 7.35 and PaCO_2_ ≥ 55 mmHg. Patients will be randomly assigned 1:1 to receive NIV or HFNT. NIV will be applied through a mask with a dedicated ventilator in pressure support mode. Positive end-expiratory pressure will be set at 3–5 cmH_2_O with inspiratory support to obtain a tidal volume between 6 and 8 ml/kg of ideal body weight. HFNT will be initially set at a temperature of 37 °C and a flow of 60 L/min. At 2 and 6 h we will assess arterial blood gases, vital parameters, respiratory rate, treatment intolerance and failure, need for endotracheal intubation, time spent under mechanical ventilation (both invasive and NIV), intensive care unit and hospital length of stay, and hospital mortality.

Based on an α error of 5% and a β error of 80%, with a standard deviation for PaCO_2_ equal to 15 mmHg and a noninferiority limit of 10 mmHg, we computed a sample size of 56 patients. Considering potential drop-outs and nonparametric analysis, the final computed sample size was 80 patients (40 per group).

**Discussion:**

HFNT is more comfortable than NIV in COPD patients recovering from an episode of exacerbation. If HFNT would not be inferior to NIV, HFNT could be considered as an alternative to NIV to treat COPD patients with mild-to-moderate respiratory acidosis.

**Trial registration:**

ClinicalTrials.gov, NCT03370666. Registered on December 12, 2017.

**Electronic supplementary material:**

The online version of this article (10.1186/s13063-019-3514-1) contains supplementary material, which is available to authorized users.

## Background

Severe exacerbation of chronic obstructive pulmonary disease (COPD) is defined as an acute worsening of chronic respiratory symptoms and decompensated respiratory acidosis [1]. In the case of hypercapnic acute respiratory failure (ARF), the sole optimization of standard medical therapy may fail in up to 74% of patients [2]. In addition to pharmacological therapy, recent guidelines recommend the use of noninvasive ventilation (NIV) in the absence of metabolic causes of acidosis [1, 3]. Indeed, NIV improves vital signs and gas exchanges [2, 4], increasing alveolar ventilation [5]. NIV also reduces the patient’s effort [6], dyspnea [2, 7], the need for intubation [2, 8, 9], the intensive care unit (ICU) length of stay [9] and the mortality rate [2, 9, 10]. However, there are several determinants for NIV success, including comfort related to the interface and patient–ventilator interaction [4, 11–18].

High-flow nasal therapy (HFNT) is a more recent treatment, which delivers a heated and humidified air–oxygen mixture at high flow (up to 60 L/min) through large-bore nasal cannula [19, 20]. Compared to standard oxygen therapy, HFNT washes out the anatomical dead space of the upper airway, generates a certain amount of expiratory pharyngeal pressure proportional to the flow, which increases the end-expiratory lung volume, slows the respiratory rate by decreasing the respiratory time [21], promotes the clearance of tracheobronchial secretions and reduces the inspiratory effort [22–24].

While a large body of evidence is already supporting the use of HFNT in critically ill patients with hypoxemic ARF [23, 25–28], few studies have evaluated its effectiveness in COPD patients [20]. In stable COPD patients, HFNT reduces the respiratory rate [29] and the arterial partial pressure of carbon dioxide (PaCO_2_) [29, 30], increases the tidal volume and the end-expiratory lung volume [29], and reduces the work of breathing and the rapid shallow breathing pattern [31, 32]. In patients recovering from an episode of hypercapnic ARF due to COPD exacerbation, the respiratory rate and drive significantly increase with standard oxygen at NIV discontinuation, while not with HFNT [33]. Furthermore, HFNT guaranteed optimal comfort [22, 34].

A recent prospective observational trial enrolling 92 patients with severe COPD exacerbation and moderate hypercapnic acute respiratory failure found no difference in 30-day mortality and the intubation rate between HNFT and NIV [34]. Currently, no randomized controlled trials have compared the efficacy of HFNT with that of NIV as first-line treatment in exacerbated COPD patients.

We conceived the present protocol in order to evaluate whether HFNT is noninferior to NIV in reducing PaCO_2_, in patients with an exacerbation of COPD and mild-to-moderate respiratory acidosis.

## Methods

### Study design

This is an investigator-initiated, unblinded, multicenter, noninferiority, randomized controlled trial. Randomization has been achieved using a computer-generated randomization sequence, generated by an independent investigator not otherwise involved in the trial, with an allocation ratio of 1:1 and with a permuted block method. A single randomization list was created for all participants. Allocation concealment has been maintained by using sequentially numbered sealed opaque envelopes. Each envelope contains the allocation of the patient to either control (NIV) or intervention (HFNT), with a unique patient identifier code. The randomization is based on a centralized phone call system. Due to the research design, neither the individual collecting data nor the patient can be blinded to treatment allocation.

**Fig. 1:**
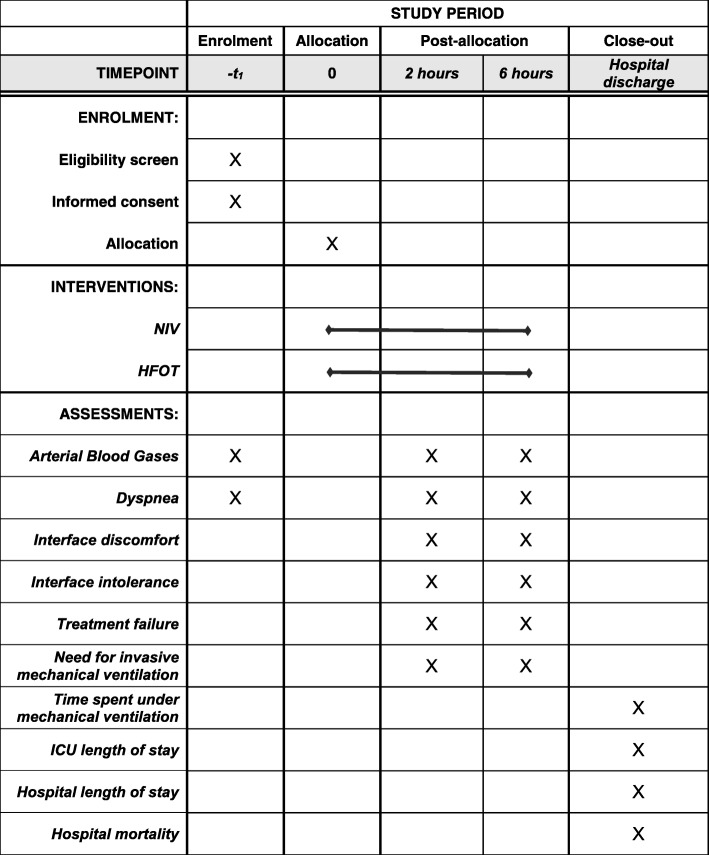
A schedule for enrolment, intervention and assessment according to the Standard Protocol Items: Recommendations for Interventional Trials (SPIRIT) indications. HFNT high-flow nasal therapy through nasal cannula, ICU intensive care unit, NIV noninvasive ventilation

**Fig. 2:**
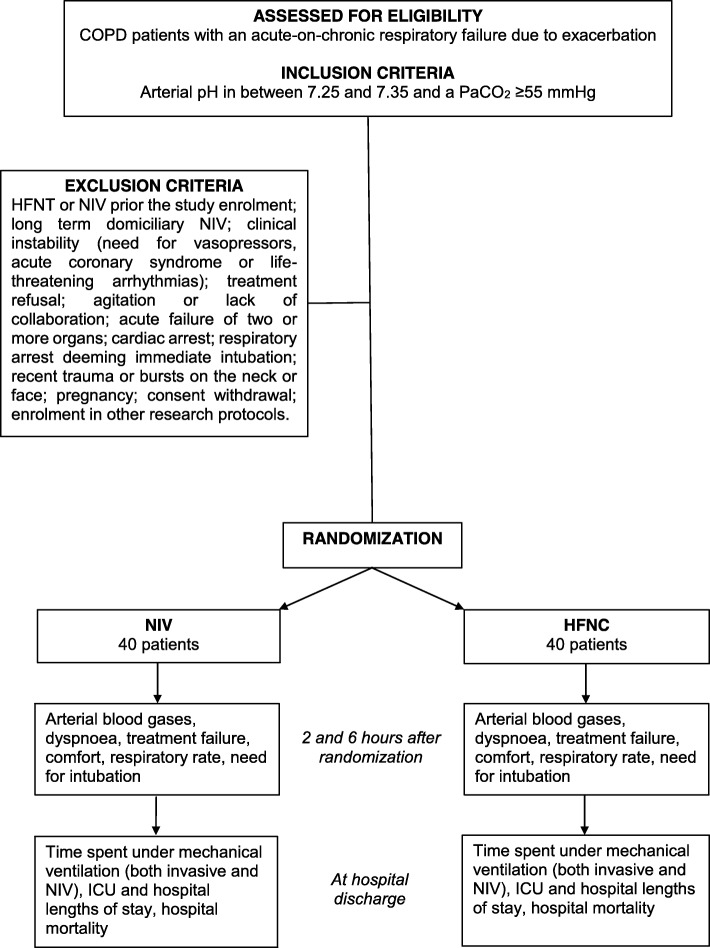
Study flow chart according to CONsolidated Standards of Reporting Trials. COPD chronic obstructive pulmonary disease, HFNT high-flow nasal therapy through nasal cannula, ICU intensive care unit, NIV noninvasive ventilation, PaCO_2_ arterial partial pressure of carbon dioxide

The study was prospectively registered with ClinicalTrials.gov in December 2017 (identifier: NCT03370666). This protocol has been designed in accordance with the Standardized Protocol Items: Recommendations for Interventional Trials (SPIRIT) guidelines and checklist (Additional file 1). A schedule for enrolment, intervention and assessment (SPIRIT figure) is outlined in Fig. 1. A CONsolidated Standards of Reporting Trials (CONSORT) diagram is shown in Fig. 2.

### Setting

The study will be conducted in the emergency department, ICU or respiratory unit of the following Italian centers: “Mater Domini” Hospital; “Magna Graecia” University, Catanzaro; Policlinico “Paolo Giaccone”, University of Palermo; University Hospital of Udine; S. Donato Hospital, Arezzo; “*S. Maria* della Misericordia” Hospital, Perugia; and “Città della Salute e della Scienza” University Hospital, Torino. The study protocol has been prospectively approved by the local ethical committees. Further centers have recently submitted the study protocol for ethical committee approval.

### Study population

We will consider eligible consecutive adult (i.e. ≥ 18 years/old) patients with a diagnosis of COPD according to the GOLD criteria [1] admitted for an acute-on-chronic respiratory failure due to exacerbation, with arterial pH between 7.25 and 7.35 and PaCO_2_ ≥ 55 mmHg, to the emergency department or ICU or respiratory unit of the participating centers. Written informed consent will be acquired before the patient’s inclusion. Patients will be excluded if they meet one or more of the following criteria: already received HFNT or NIV, prior to study enrolment; long-term domiciliary NIV; clinical instability, as defined by the need for vasopressors, acute coronary syndrome or life-threatening arrhythmias [35]; treatment refusal; agitation, as defined by Richmond Agitation Sedation Scale (RASS) score ≥ 2, or lack of collaboration, as defined by Kelly Matthay score ≥ 5 [35]; acute failure of more than two organs [35]; cardiac arrest; respiratory arrest deeming immediate intubation; recent trauma or bursts on the neck or face; pregnancy; consent withdrawal; and enrolment in other research protocols. The study flow chart is shown in Fig. 2.

### Intervention group

Patients randomized to the intervention group will receive HFNT (Optiflow and MR850 or AIrVO_2_™; Fisher & Paykel Healthcare, Auckland, New Zealand), initially set at 60 L/min and a temperature of 37 °C. In the case of discomfort, the flow and/or temperature will be downregulated to the most tolerated setting. In the HFNT group, a rescue NIV treatment will be allowed in the case of worsening of respiratory failure and/or treatment failure, before proceeding with an eventual endotracheal intubation and invasive mechanical ventilation.

### Control group

Patients randomized to the control group will receive NIV applied through a total full-face or oro-nasal mask. The ventilator will be set in pressure support ventilation (PSV) mode, with positive end-expiratory pressure (PEEP) between 3 and 5 cmH_2_O. The inspiratory pressure support will be regulated in order to achieve a measured or estimated tidal volume equal to 6–8 ml/kg of ideal body weight [35]. The treating physicians, based on local availability, will choose the ventilators used to deliver NIV.

In both groups, treatment other than ventilatory support will be supplied according to current guidelines [1]. Sedatives will be allowed to improve the patient’s comfort and tolerance of the interfaces. The inspired oxygen fraction (FiO_2_) will be set to maintain a peripheral saturation (SpO_2_) target between 88 and 92% [1]. In the case of treatment interruption due to any reason, patients will receive oxygen supply through a Venturi mask to achieve the same SpO_2_ target. During study interventions, patients will be monitored by at least continuous SpO_2_, electrocardiogram and noninvasive blood pressure.

### Study endpoints

The primary endpoint of the study will be to evaluate whether HFNT is noninferior to NIV in reducing PaCO_2_, at 2 h after the group allocation, in patients with exacerbation of COPD and mild-to-moderate respiratory acidosis. The secondary endpoints will be: noninferiority of HFNT to NIV in reducing PaCO_2_ at 6 h after randomization; dyspnea score; treatment failure rate; discomfort related to the interface or to symptoms related to airway and mouth dryness; respiratory rate; need for endotracheal intubation; time spent under mechanical ventilation (both invasive and NIV); ICU and hospital length of stay; and hospital mortality.

### Data collection and outcome assessment

For each patient, we will collect anthropometric data (age, gender, weight and height) and baseline characteristics (the Simplified Acute Physiology Score (SAPS II), the Kelly-Matthay score [36], the Charlson index [37] and the Richmond Agitation Sedation Scale (RASS) score [38]). Furthermore, soon before randomization we will record vital parameters (heart rate, blood pressure, breath frequency and temperature), the amount of secretions, the presence of dyspnea (as reported by the patient through the Borg scale) [39] and arterial blood gases (ABGs) at patient inclusion.

At 2 and 6 h after the patient’s randomization to receive NIV or HFNT, we will collect all vital parameters, ABGs, the RASS score, the Kelly-Matthay score [36], the grade of dyspnea and the need for sedatives. Sedation will be allowed to increase tolerance to the treatment [40, 41]. Moreover, we will record: the settings of NIV and HFNT; the discomfort related to the interface, as assessed through a 11-point Numeric Rating Scale (NRS) [15, 42, 43]; and the intolerance to the interface due to flow, temperature, noise, claustrophobia, sweating, tightness, airway dryness, vomiting gastric distension, ocular irritation or skin breakdown, separately [42, 44, 45].

We will also record: the rate of treatment intolerance, as defined by the inability of the patient in continuing NIV or HFNT treatment; the rate of treatment failure, as defined by the presence of PaCO_2_ worsening or reduction < 10 mmHg from baseline assessment, or worsening or no improvement of dyspnea, or respiratory rate > 30 breaths/min; need for endotracheal intubation; time spent under mechanical ventilation (both invasive and NIV); ICU and hospital length of stay; and hospital mortality.

The decision to intubate the patient will be taken by the physician in charge of the patient, in the case of the presence of one of the following criteria: respiratory arrest, respiratory apnea or pauses with loss of consciousness, severe agitation, bradycardia (< 50 beats/min) with loss of consciousness, hemodynamic instability with systolic arterial pressure < 70 mmHg, need for invasive mechanical ventilation due to worsening in arterial blood gases and pH, need for suctioning due to unmanageable secretions or intolerance to all of the interfaces (including shift of treatment to NIV in the HFNT group).

Data will be collected on a dedicated case report form that will be sent to the data manager in de-identified form. The data manager will be responsible for entering data into a dedicated database, to code and store them. Treatment allocation will also be processed in order for the independent investigator in charge of the statistical analysis to be blinded.

### Statistical analysis

For the purpose of the study, we computed a sample size of 56 patients, given an α error of 5% (one-sided) and a β error of 80%, with a standard deviation for the primary outcome equal to 15 mmHg and a noninferiority limit of 10 mmHg [35]. After considering potential drop-outs (30%) [35] and an increase in sample size for nonparametric analysis (15%), the final computed sample size was 80 patients (40 per group).

An independent investigator, blinded to treatment allocation, will undertake all statistical analyses on an intention-to-treat basis. After checking the Gaussian distribution, continuous data will be presented as mean (standard deviation) or median (25th–75th IQR).

Categorical data will be expressed as count and percentage. Continuous data will be compared by the Mann–Whitney *U* test or the Student *t* test, and categorical data by the chi-square test or the Fisher exact test [46]. The Kruskal–Wallis test will be used to check between-group differences at baseline, 2 h and 6 h. Friedman’s test or repeated-measures ANOVA will be used to check within-group differences at different time points. *p* < 0.05 will be considered significant.

### Study organization

The steering committee consists of two principal investigators (AC, FL) plus six members with recognized expertise on NIV/HFNT in COPD patients. The steering committee invited other centers based on expertise and availability of study patients and materials. Each participating center has a local principal investigator in charge. They act as the guarantors of the data, and are responsible for data management and storage in their centers. Local principal investigators also communicate with the principal investigators and transfer patient data in an anonymized form on standard case report forms; in the case of logistic difficulties or study doubts, the local investigators can ask for immediate phone call support from principal investigators, to achieve adequate participant enrolment. Data monitoring will be done by an ndependent investigator serving as the data manager. This will be done by means of queries on the final databases, after collecting the case report forms from enrolling centers, to check for abnormalities and inconsistencies.

## Discussion

HFNT is increasingly used in different settings [23, 25–27]. Some studies indicate that HFNT has physiological advantages in both stable and exacerbated COPD patients [20]; in particular, in patients recovering from an episode of hypercapnic ARF due to COPD exacerbation, HFNT reduces the respiratory effort and rate no differently from NIV, while it maintains similar gas exchanges and improved comfort [22]. A recent observational trial found no difference between NIV and HNFT in terms of 30-day mortality and intubation rate in severe exacerbation of COPD [34].

Currently, NIV is strongly suggested as standard treatment for exacerbated COPD patients with respiratory acidosis [3]. However, NIV is affected by several drawbacks such as reduced comfort and poor patient–ventilator interaction and synchrony, which is often difficult to be recognized and managed [4, 11–13, 16]. We therefore design this randomized controlled trial to assess the noninferiority of HFNT in reducing the PaCO_2_, as compared to NIV. In fact, whenever the trial demonstrates a noninferiority of HFNT compared to NIV, the physician may use HFNT as an alternative ventilatory support in exacerbated COPD patients with mild-to-moderate respiratory acidosis. This would also allow the possibility to use another support with a different interface in a rotational strategy to improve the patient’s comfort and tolerance to the treatment [47, 48]. This trial has limitations. First, blinding is not possible due to the nature of the interventions. Second, although recruitment centers have established experience in the use of ventilator support in this patient population, different practices in the use of both NIV and HNFT may influence the effect of the interventions. However, recruitment centers have established experience in the use of ventilator support in this patient population. Third, the study is a noninferiority trial with a physiologic outcome, leaving uncertainty on stronger patient-related outcomes. It is anticipated that the results of this study will be used to design further randomized clinical trials powered to assess major clinical outcomes.

## Trial status

The study is currently recruiting patients. The protocol was approved by the Ethics Committee of the coordination center on 24 October 2017 (“Comitato Etico sezione Area Centro—N°245”). The first patient was enrolled on 13 March 2018. Recruitment is expected to be completed within June 2020.

## Additional file

Additional file 1: SPIRIT 2013 Checklist: Recommended items to address in a clinical trial protocol and related documents (DOC 122 kb)

## Data Availability

The authors will share all of the individual participant data collected during the trial, after de-identification, to researchers who provide a methodologically sound proposal.

## Abbreviations

ABG: Arterial blood gas
ARF: Acute respiratory failure
CONSORT: CONsolidated Standards of Reporting Trials
COPD: Chronic obstructive pulmonary disease
HFNT: High-flow nasal therapy
NIV: Noninvasive ventilation
NRS: Numeric Rating Scale
PaCO_2_: Arterial partial pressure of carbon dioxide
PEEP: Positive end-expiratory pressure
RASS: Richmond Agitation Sedation Scale
SAPS II: Simplified Acute Physiology Score
SPIRIT: Standard Protocol Items: Recommendations for Interventional Trials
